# Diabetic Cardiomyopathy: An Update on Emerging Pathological Mechanisms

**DOI:** 10.2174/011573403X331870241025094307

**Published:** 2024-11-04

**Authors:** Chirag Kakkar, Veerta Sharma, Ashi Mannan, Gaurav Gupta, Sachin Singh, Puneet Kumar, Kamal Dua, Amarjot Kaur, Shareen Singh, Sonia Dhiman, Thakur Gurjeet Singh

**Affiliations:** 1 Chitkara College of Pharmacy, Chitkara University, Rajpura, Punjab, India;; 2 Centre of Medical and Bio-allied Health Sciences Research, Ajman University, Ajman, 346, United Arab Emirates;; 3 Lovely Institute of Technology (Pharmacy), Lovely Professional University, Phagwara, Punjab, India;; 4 Discipline of Pharmacy, Graduate School of Health, University of Technology Sydney, Broadway, P.O. Box 123, Ultimo, NSW, 2007, Australia;; 5 Department of Pharmacology, School of Pharmaceutical Sciences, Central University of Punjab, Ghudda, Bathinda, India;; 6 Faculty of Health, Australian Research Centre in Complementary and Integrative Medicine, University of Technology Sydney, Ultimo, NSW, 2007, Australia

**Keywords:** Diabetic cardiomyopathy, pathology, pharmacological approach, diabetes, oxidative stress, calcium

## Abstract

Diabetic Cardiomyopathy (DCM) is a notable consequence of diabetes mellitus, distinguished by cardiac dysfunction that occurs separately from coronary artery disease or hypertension. A recent study has revealed an intricate interaction of pathogenic processes that contribute to DCM. Important aspects involve the dysregulation of glucose metabolism, resulting in heightened oxidative stress and impaired mitochondrial function. In addition, persistent high blood sugar levels stimulate inflammatory pathways, which contribute to the development of heart fibrosis and remodelling. Additionally, changes in the way calcium is managed and the presence of insulin resistance are crucial factors in the formation and advancement of DCM. This may be due to the involvement of many molecular mechanistic pathways such as NLRP3, NF-κB, PKC, and MAPK with their downstream associated signaling pathways. Gaining a comprehensive understanding of these newly identified pathogenic pathways is crucial in order to design precise therapy approaches that can enhance the results for individuals suffering from diabetes. In addition, this review offers an in-depth review of not just pathogenic pathways and molecular mechanistic pathways but also diagnostic methods, treatment options, and clinical trials.

## 1 INTRODUCTION

Diabetes is a complicated endocrine disorder characterized by hyperglycemia resulting from insulin deficiency, insulin resistance, or both, and it interacts with several different susceptibility alleles as well as environmental factors [1, 2]. The health of hundreds of millions is at risk due to the chronic metabolic condition known as diabetes. According to the International Diabetes Federation, about 537 million persons aged 20-79 had diabetes in 2021, with this figure expected to rise to 783 million by 2045 if present trends continue. According to the World Health Organization (WHO), more than 50% of deaths from diabetes individuals are caused by diabetic cardiovascular diseases (DCvds). DCvds include diabetic cardiomyopathy (DCM), heart failure, cerebrovascular disease, and coronary heart disease [3, 4]. DM persons have a two-fold increased chance of developing heart failure when comparing all DM sequelae, with DCM accounting for over 80% of death cases [5, 6]. There is a number of epidemiological evidence to back up the link between DM and heart failure. This increased risk of congestive heart failure is determined by analyzing multiple factors, including age, weight, blood pressure, lipid profile, and coronary artery disease. Comparing diabetic men to their non-diabetic counterparts, it is found that the likelihood of developing cardiac failure is enhanced by a factor of two. Additionally, the incidence of developing cardiac failure is five folds higher in diabetic women. Additionally, compared to non-diabetics, people with diabetes have considerably higher mortality rates. DCM is described as a distinct condition in diabetic people with cardiac anatomical abnormalities and dysfunctions that are unrelated to uncontrolled hypertension, severe valve disease, and heart disease [7]. However, there is no commonly accepted definition and reliable epidemiological data on death [8]. Patients with obesity, dyslipidemia, and insulin resistance are more likely to develop metabolic cardiomyopathy, also known as insulin resistance-induced cardiomyopathy or lipotoxic cardiomyopathy, even if they do not have diabetes [9, 10]. In 1972, diabetes cardiomyopathy was identified for the first time through post-mortem pathological examinations of four diabetic patients exhibiting signs of heart failure. Autopsy finding of these patients shows hypertrophy of the left and right ventricular as well as cardiac fibrosis [11, 12]. There are several probable clinical and molecular pathways involved in the pathogenesis of diabetic cardiomyopathy. In insulin resistance, reduced glucose absorption leads to increased fatty acid oxidation for energy [13]. This enhanced fatty acid oxidation results in the buildup of diacylglycerol (DAG) and ceramides (toxic lipid byproducts) in heart cells [14]. DAG and ceramides activate the PKC pathway, which impairs glucose absorption and contributes to insulin resistance [15]. This causes metabolic dyshomeostasis, which adds to myocardial energy shortages, culminating in cardiac dysfunction and structural changes, ultimately leading to DCM [16]. Furthermore, insulin resistance results in hyperinsulinemia due to a compensatory mechanism. Hyperinsulinemia promotes the PI3K/Akt signalling pathways, which regulate cellular growth and survival [17]. Therefore, overstimulation of these pathways leads to cardiac hypertrophy, maladaptive remodelling leading to myocardial fibrosis, stiffness, and, finally, loss of heart function as characterised in DCM [18]. Moreover, it has been observed that visceral fats, which have high metabolic activity, might enhance the inflammatory response by releasing several bioactive molecules, such as adipokines, including TNF-α, IL-6, and resistin [19]. The presence of these inflammatory mediators disrupts the normal functioning of the insulin signalling system, leading to a condition of insulin resistance through the PI3K/Akt signalling pathway [20]. Visceral fat is a provider of free fatty acids, and their release is heightened in a condition of insulin resistance. This, in turn, contributes to lipotoxicity in the myocardium and leads to the development of DCM [21]. Moreover, chronic hyperglycemia and insulin resistance lead to endoplasmic reticulum stress, calcium dyshomeostasis, mitochondrial dysfunction, oxidative stress, activation of the Renin-Angiotensin-Aldosterone System (RAAS), and various other pathologic factors were linked to the progression and development of DCM [22-25]. The development of diabetic cardiomyopathy is also influenced by the NLRP-3 inflammasome, MAPK, NF-κB, and other molecular pathways, with an emphasis on both well-established and novel or developing processes. DCM is diagnosed by detecting the alterations that occur in cardiac cells using several non-invasive methods, such as imaging instruments, Magnetic resonance imaging, echocardiography, and minimally invasive diagnostic biomarkers [5, 26]. Hence, the aim of this review is to offer a thorough understanding of the developing pathogenic mechanisms implicated in diabetic cardiomyopathy (DCM). The review seeks to elucidate the fundamental causes of this devastating heart disease by analysing the complex interaction of components such as dysregulated glucose metabolism, oxidative stress, inflammation, and changes in calcium handling. Acquiring this knowledge is essential for recognising possible therapy targets and formulating efficient methods to enhance clinical outcomes for individuals with diabetes.

## 2 STAGES OF DIABETES CARDIOMYOPATHY

DCM consists primarily of two main parts: the first is short-term, during which various metabolic changes occur in the body, and the body adapts physiologically to these metabolic changes; the second part involves degenerative changes for which the myocardium has a limited capacity to repair these degenerative changes. Thus, treatment or therapies administered during the earliest stages of diabetes can result in a delay in the disease's progression.

### 2.1 Early Stage

Hyperglycemia initiates DCM, which is characterized by an elevated amount of free fatty acids (FFA), the depletion of glucose transporters (GLUT-1 and -4), alterations in calcium (Ca^2+^) homeostasis, and insulin resistance at the molecular level. The early stage is predominantly asymptomatic, and the heart becomes hypertrophic. Alterations in cardiac structure, such as LV size, the thickness of the heart wall, and mass, are negligible [27]. Several functional alterations occur, such as diastolic dysfunction. Endothelial dysfunction is most prevalent in the initial stages of DCM [28].

### 2.2 Middle Stage

Numerous cellular changes occur at this stage, including impairment in the transport of calcium ions and defect in fatty acid metabolism, which leads to an increase in levels of tumor growth factor 1 (TGF-1), RAAS system, apoptosis, and necrosis of cardiac myocytes, as well as myocardial fibrosis, which eventually leads to injury in myocytes and a decrease in ejection fraction. At this point, advanced glycation end products (AGE) and insulin resistance start to emerge, and endothelial dysfunction is not significant [27]. Increased LV size, wall thickness, and mass, as well as diastolic dysfunction and minor alterations in systolic functions, cause an ejection fraction of less than 50% [29].

### 2.3 Last Stage

As DCM progresses from the middle to the late stages, changes in myocardial fibrosis result in an increase in disease severity, which leads to myocardial microvascular change that affects both systolic and diastolic functions [28]. At this stage, both structural and functional myocardial microvascular alterations occur, as well as recurrent microvascular spasms. There is a rise in LV size, mass, cardiac fibrosis, wall thickness, and microvascular blood vessel disorders. The last stage of DCM is usually accompanied by an increase in blood pressure (hypertension) and the onset of ischemic heart disease in diabetes [27].

## 3 PATHOLOGICAL MECHANISMS OF DIABETIC CARDIOMYOPATHY (DCM)

### 3.1 Endoplasmic Stress

It plays essential roles in numerous processes, which include the synthesis and distribution of steroids and phospholipids; synthesis, folding, modification, and transport of proteins; and Ca^2+^ storage within its lumen as well as their controlled release in the cytoplasm [30]. In the endoplasmic reticulum (ER) lumen, nascent polypeptides undergo posttranslational modifications and acquire their respective functions. Those proteins that undergo post-translational modifications correctly leave the ER, whereas those that do not are degraded by proteasomes [31]. Multiple factors, such as folding enzymes, molecular chaperones, and Ca^2+^ ions, contribute to the efficient functioning of the ER. Several factors, such as ischemia, radiations, cardiac oxidative stress, disruption of Ca^2+^ homeostasis, lipotoxicity, and enhanced expression of normal as well as improperly folded proteins, cause these proteins to accumulate in an unfolded state that disrupts the homeostasis of ER. This process, known as ER stress, also activates the unfolding protein response (UPR) [7]. The UPR has two objectives: first, it attempts to restore normal cell function by protein translation inhibition and activating the signal transduction pathways that result in an upsurge in the development of molecular chaperones responsible for protein folding.; second, if these objectives are not accomplished within a predetermined time frame or if the disruption is sustained, the UPR activates the apoptotic pathway, which causes the degradation of damaged and misfolded protein by ER-associated degradation (ERAD) complex, which ultimately results in an increase in cardiac cell apoptosis and autophagy that will lead to DCM. Thus, UPR can be regarded as a protective mechanism for lipid biosynthesis, Ca^2+^ storage and signaling, and the synthesis of proteins along with their posttranslational modifications, folding as well as secretion [30]. The UPR is stimulated by misfolded protein accumulation in three signaling pathways: the inositol-requiring enzyme-1 (IRE-1), Activating transcription factor-6 (ATF-6), and protein kinase RNA-like ER kinase pathways (PERK) [32]. These 3 signaling pathways can individually cause cell apoptosis by stimulating the executors of apoptosis, including caspase 12, Jun N-terminal kinase (JNK), and ATF-4 [33]. Multiple canonical UPR pathways can be activated by ER stress to induce autophagy. Ca^2+^ release from the ER can activate numerous kinase enzymes which control autophagy. Ca^2+^/calmodulin-dependent kinase (CaCMKK) phosphorylates and activates AMP-activated protein-kinase (AMPK), which subsequently hinders the mammalian target of rapamycin complex-1 (mTORC-1). mTORC1 regulates autophagy by suppressing the Atg1-13-101/FIP200 complex, therefore, mTORC1 inhibition encourages the initiation of autophagy. Hyperglycemia-mediated development of monocyte chemoattractant protein-1 (MCP-1) and the induction of MCP-1-induced protein (MCPIP) results in the stimulation of UPR. Stimulated UPR upregulates IRE1, leading to the activation of JNK and, eventually, autophagy. Prolonged autophagy leads to cardiac cell apoptosis, resulting in DCM [7]. Several non-clinical studies have revealed the significance of ER stress in DCM. A study conducted by Wu and colleagues demonstrated that Valsartan, an AT1 receptor antagonist, ameliorates ER stress-induced myocardial cell death and cardiac remodeling in DCM [34]. Guo and colleagues examined the correlation between ER stress-mediated Sirtuin-1 and apoptosis in H9C2 cardiomyocytes. The findings demonstrated that SIRT-1 inhibits cardiomyocyte apoptosis through PERK/eIF2α and IRE1α/JNK mediated pathways [35]. Administration of ginsenoside Rg1 in STZ-induced diabetic rats attenuated myocardial damage. It markedly lowered ER stress-induced cardiomyocyte apoptosis *via* the dose-dependent reduction in the expression of cleaved caspase 12 protein and GRP78 [36]. Astragalus polysaccharides, the primary bioactive component obtained from *Astragalus membranaceous* decrease apoptosis of cardiomyocytes in HG-induced H9C2 cells and in STZ-induced DCM rats by blocking PERK-ATF6-CHOP signaling pathway [33]. Therefore, the data presented above implies that ER stress is a substantial contributor to DCM.

ER stress also contributes to the advancement of disease as ER serves as a main site for storing Ca^2+^ within cells [37]. In cases of DCM, ER stress disrupts the regulation of Ca^2+^, causing abnormal release of Ca^2+^ into the cytoplasm and resulting in an imbalance of Ca^2+^ levels, known as Ca^2+^ dyshomeostasis [38]. The elevated levels of Ca^2+^ in the cytoplasm cause an excessive accumulation of Ca^2+^ in the mitochondria, which in turn leads to impaired function of the mitochondria and ultimately results in oxidative stress [39]. Hence, ER stress-derived mitochondrial dysfunction and oxidative stress contribute to the progression of DCM.

### 3.2 Impaired Ca^2+^ Homeostasis

Abnormal homeostasis of Ca^2+^ ions and impairment in Ca^2+^ signaling in cardiomyocytes is the mechanistic hallmark of DCM. Ca^2+^ homeostasis must be regulated and maintained for the adequate functioning and development of the heart [40]. Calcium plays a vital role in every cycle of heart contraction and relaxation and is responsible for maintaining normal cardiac function [41]. When the action potential approaches the cardiac myocyte, depolarization of the cell membrane occurs, which results in an opening of L-type Ca^2+^ channels (LTCC). Ca^2+^ influx occurs in cardiomyocytes, which then interacts with ryanodine receptors (RyR) and triggers the release of large quantities of Ca^2+^ ions from the sarcoplasmic reticulum. This will increase the intracellular concentrations of Ca^2+^ ions surrounding the sarcomere. Ca^2+^ ions diffuse through cytosolic space and reach contractile proteins, where they interact with troponin C. Ca^2+^ binding to troponin C results in the interaction of actin and myosin filaments, which ultimately leads to myocardial contraction. Removal of Ca^2+^ ions from the cytoplasm causes the relaxation of cardiac cells. Various mechanisms are used to remove the Ca^2+^ ions, including the sarcoplasmic reticulum Ca^2+^ ATPase pump (SERCA2a); the plasma membrane Ca^2+^_ATPase_ (PMCA); the Na^+^/Ca^2+^ exchanger (NCX) and the extrusion of Ca^2+^ ions in mitochondria (Al Kury, 2020). Ca^2+^ enters mitochondria and aids multiple enzymes involved in the TCA cycle (tricarboxylic acid cycle) for the generation of ATP [41]. In the type 2 diabetes model, a substantial decline in the Ca^2+^ transient caused by a reduced influx of Ca^2+^ ions as a result of reduced expression of LTCC, decline in the sarcoplasmic Ca^2+^ level due to reduced expression of SERCA2a, enhanced expression of phospholamban; as well as impaired activity of RyR [42]. It has been investigated in a study that Phospholamban (PLB) (a protein that regulates the Ca^2+^ pump in cardiac muscle cells) inhibits the activity of SERCA2a, which appears to play a vital role in the development of DCM. As the activity of SERCA2a decreases, Ca^2+^ ions sequestration in the sarcoplasmic reticulum reduces, resulting in an elevation in Ca^2+^ ions concentration in the cytoplasm, which inhibits the relaxation of the cardiomyocyte, resulting in diastolic dysfunction [29]. Ca^2+^ homeostasis, contractile dysfunction, and diastolic dysfunction in diabetic mice can be improved by the overexpression of SERCA2a, which improves myocardial contractility in DCM [43]. In addition to this altered L-type Ca^2+^ channel activity, RyR2 and NCX are also responsible for maintaining Ca^2+^ homeostasis; therefore, targeting these proteins along with SERCA2a improves cardiac cell contractility in DCM [44]. Thus, the aforementioned evidence supports the notion that Ca^2+^ dyshomeostasis plays a crucial role in the advancement of DCM. In addition, this imbalance in Ca^2+^ levels might result in mitochondrial dysfunction, which in turn can accelerate the onset of DCM. The elevated levels of Ca^2+^ in the cytosol are transported into the mitochondria through the mitochondrial calcium uniporter (MCU) [45]. This increase in Ca^2+^ disrupts the normal functioning of the mitochondria and activates various calcium-dependent enzymes, such as pyruvate dehydrogenase (PDH) and enzymes involved in the tricarboxylic acid (TCA) cycle [46]. These enzymes are essential for ATP production, but their overactivation results in the production of reactive oxygen species (ROS) as a byproduct of increased metabolic activity and thus leads to mitochondrial dysfunction.

### 3.3 Mitochondrial Dysfunction

**Fig. (1):**
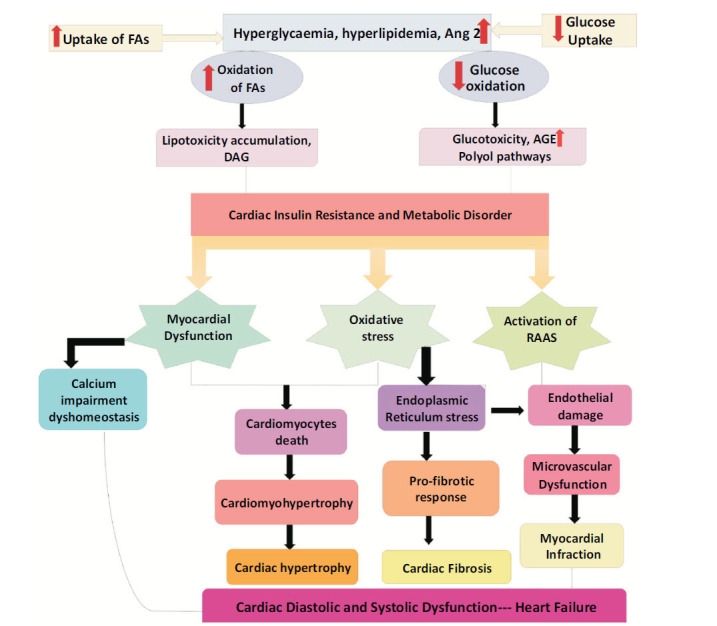
Mechanisms underlying the pathophysiology of DCM. Hyperglycemia and insulin resistance induce increases in AGEs, and lipo-toxicity, which, in turn, result in oxidative stress, mitochondrial dysfunction, and activation of RAAS. These pathophysiological abnormalities are associated with heart failure.

Mitochondrial dysfunction plays a significant role in DCM development. Mitochondria are the primary sites of both glucose and fatty acid metabolism; therefore, they are more susceptible to diabetes-related metabolic impairment [47]. Mitochondria are the primary generators of Reactive oxygen species (ROS). In tissues with high respiration rates, such as cardiomyocytes, approximately 90% of basal cellular ROS is produced by mitochondria. ROS generated by the mitochondria can alter numerous physiological pathways. In addition to directly oxidizing proteins, ROS can also oxidize lipids to generate lipid peroxidation products that can damage proteins or phospholipids [48, 49]. Impairment in the functioning of mitochondria leads to overproduction of ROS, which ultimately causes the death of cardiac cells and results in DCM. In cardiomyocytes, oxidative phosphorylation of glucose in the mitochondria is responsible for 90% of ATP production; however, in diabetes, FFA are oxidized in the mitochondria to generate ATP. Oxidation of FFA causes a rise in ROS production, which disrupts the functioning of mitochondria, resulting in cardiac cell death [50, 51]. Since the heart has a lower level of antioxidants than other organs, oxidative stress predominantly affects cardiac cells, resulting in cell damage. When diabetes develops, different antioxidants are initially upregulated as a compensatory response to oxidative stress. Still, later, diabetes induces ROS and RNS production and decreases the antioxidant activity in the heart [52]. In cells, numerous genes are present in antioxidant response elements (AREs), which code for proteins that act as antioxidants to neutralize ROS. Nuclear factor erythroid factor 2-related factor (Nrf-2) is essential for the expression of protective antioxidant genes, which induces the activation of numerous antioxidants [52]. A transgenic mouse model that expresses the human MT-IIA gene, particularly in cardiomyocytes, has been developed, and it has been proven that metallothioneins (MT) function as an antioxidant in the heart, quenches all free radicals, and protect cardiac cells from oxidative stress induced by ischemia/reperfusion, acute or chronic doxorubicin treatment, and Cu deficiency. MT in the heart can defend against early-stage diabetes-induced myocardial damage and late-stage cardiac dysfunction, thereby aiding in the prevention of DCM [53]. In diabetic patients, however, the expression of cardiac Nrf2 decreases, causing a reduction in the quenching of ROS and RNS, which causes an upsurge in oxidative stress and the progression of DCM. The upregulation of the Nrf2 protein or Nrf2 inducers has a protective effect against DCM (Fig. **1**) [52].

### 3.4 Activation of RAAS

The RAAS controls extracellular volume, arterial blood pressure, and plasma sodium content, making it a vital system of the human body. Angiotensinogen is transformed into angiotensin-1 by renin, which is then transformed into angiotensin-2 (Ang-2) by angiotensin-converting enzyme (ACE). Ang-2 is an active component of the RAAS that exerts its effects on angiotensin receptors [54]. Most of the actions of Ang-2 are believed to be primarily mediated through the activation of angiotensin-2 type-1 (AT1) receptors. Ang-2 has a wide range of biological activities, including salt retention, which raises arterial blood pressure, aldosterone production, strong vasoconstrictor effects, and interactions with the sympathetic nervous system to enhance muscle tone. Pathophysiological actions of Ang-2 include cardiomyocyte hypertrophy, left ventricular hypertrophy (LVH), cardiac fibrosis, proliferation and hypertrophy of vascular smooth muscle cells, and cardiomyocyte apoptosis [55]. Patients with diabetes had myocardial cells with intracellular Ang II levels 3.4 times more than those of non-diabetics [56]. In states of insulin resistance, increased activation of the RAAS has a significant impact on the pathogenesis of diabetic cardiomyopathy [50]. Due to RAAS activation, cardiomyocytes undergo oxidative stress, apoptosis, or necrosis, leading to an increase in interstitial fibrosis [57]. Aldosterone and Ang-2 can stimulate mitogen-activated protein-kinase (MAPK), which stimulates the proliferation of fibroblasts while causing cardiomyocyte fibrosis and apoptosis. Additionally, both aldosterone as well as Ang-2 induce considerable oxidative stress in cardiomyocytes through the transactivation of nicotinamide adenine dinucleotide phosphate (NADPH) oxidase and the generation of ROS [58]. A rise in oxidative stress damages cardiomyocyte cells, leading to apoptosis and cell death [59]. Aldosterone can directly cause cardiac fibrosis by triggering pro-inflammatory factors that result in matrix metalloproteinase (MMP) activation, and increased collagen and elastin deposition. The cardiomyocyte mineralocorticoid receptor (MR) is believed to play a role in mediating some actions of aldosterone [60]. The binding of aldosterone to MR initiates the replacement of myofibroblasts by activating ERK1/2, which promotes the proliferation of fibroblasts and stimulates the MAPK pathway. Moreover, aldosterone increases transforming growth factor-β (TGF-β) (stimulator of fibrosis); plasminogen activator inhibitor-1 (PAI-1), a crucial factor involved in fibrinolysis; and extracellular matrix (ECM) protein, leading to enhanced remodeling [61]. In DCM, antagonizing the action of aldosterone reduces hypertrophy in cardiomyocytes, thereby improving myocardial function. Antagonizing or inhibiting the RAAS pathway decreases MMP-2 activity, TGF expression, and MAPK signaling, thereby ameliorating cardiac dysfunction [41]. In STZ-induced diabetic C57/ bl6 mice, Irbesartan, an antagonist of the AT-1 receptor, normalized the activity of MMP-2 and decreased LV dysfunction, cardiac failure as well as cardiac fibrosis [62]. Administering an ethanolic extract of *Cissus quadrangularis* in STZ and high-fat diet-induced DCM by decreasing the production of ROS and cardiac inflammation through inhibition of the RAAS system [63]. Administration of ACE inhibitor captopril to STZ-induced diabetic rats has shown cardioprotective effect by reducing ROS production. LCZ696 (Valsartan/ Sacubitril), the first clinically used ACE inhibitor, reduced oxidative stress and prevented cell death in STZ-induced cardiac diabetes while improving ventricular remodeling and cardiac function (Fig. **1**) [64].

### 3.5 Altered Metabolism

Alterations in the myocardial substrate and energy metabolism, along with increased fatty acid absorption, significantly contribute to the emergence of DCM. In normal physiological conditions, the heart's metabolic flexibility allows it to use both fatty acids and glucose as a source of energy. Fatty acid translocase (FAT) increases fatty acid absorption, whereas glucose uptake is mediated by GLUT-4 [50, 65]. In general, consistent ATP production is closely related to the proper oxidation of fatty acids and glucose in response to the constant demand of the heart. Due to hyperlipidemia and insulin resistance, diabetic myocytes utilize more free fatty acids and less glucose as a metabolic substrate. Peroxisome proliferator-activated receptor (PPAR), a nuclear receptor transcription factor, is activated in cardiomyocytes by the over-expression of essential enzymes as a consequence of a rise in myocardial fatty acid levels [66, 67]. PPAR-α regulates the transcription of multiple genes involved in myocardial fatty acid utilization and oxidation [68]. Activation of the PPAR receptor increases the expression of pyruvate dehydrogenase, which reduces glucose oxidation. Malonyl CoA normally inhibits the enzyme carnitine palmitoyl transferase-1, which is responsible for preventing fatty acid oxidation.

PPAR activation increases CD36 gene expression, which regulates cellular fatty acid uptake and increases malonyl CoA decarboxylase (MCD). MCD degrades malonyl CoA, thus depressing carnitine palmitoyltransferase-1 and promoting mitochondrial FA uptake and oxidation [57]. The oxidation of fatty acids leads to ROS accumulation, which reduces the myocardium's ability to oxidize fatty acids. This reduction in the oxidative capacity leads to the accumulation of lipids in the presence of sustained accelerated fatty acid uptake, thereby eliciting the deleterious effects associated with lipotoxicity [68-70]. Lipid accumulation leads to the production of lipid intermediates like ceramide, which encourages cardiomyocyte apoptosis and contractile dysfunction [57]. PPAR-α overexpression by the elevated intracellular fatty acid in the heart increases fatty acid uptake and oxidation. Therefore, it is believed PPAR-α regulates the metabolism of fatty acids in the heart. Inactivating PPAR-α in the heart eliminates the upregulation of fatty acid metabolic genes and switches the substrate from fatty acid to glucose [71]. In PPAR-α deficient animal models, fatty acid oxidation and fatty acid utilization pathways were found to be reduced [66]. PPAR-β is also abundantly expressed in the cardiac tissue, which is activated by an increased intracellular fatty acid, and they augment the expression of a certain group of genes that promotes fatty acid utilization. Another type of PPAR is PPAR-γ, which is predominantly expressed in adipose tissues and is barely detectable in cardiomyocytes and stimulation of PPAR-γ promotes the expression of lipogenic genes and controls lipogenesis. PPAR-γ agonists decrease the plasma lipid levels in the obese Zucker diabetic fatty rats, which further reduces cardiac ceramide and, improves heart function and reversed lipotoxicity [71-73]. Reduction in ATP production is linked to elevated fatty acid oxidation in obese-induced diabetic mice, suggesting decreased cardiac mitochondrial efficiency. It has been shown that elevated fatty acid oxidation in diabetic hearts is related to higher myocardial oxygen consumption, which is not followed by an equal rise in myocardial contractility, resulting in decreased cardiac efficiency [74].

### 3.6 Oxidative Stress

In the diabetes mellitus state, increased ROS stimulated by high glucose is regarded as a significant factor in the development of DCM. Excessive absorption of Ca^2+^ from the cytosol causes mitochondrial malfunction, which disrupts the electron transport chain (ETC) and overactivates enzymes crucial for ATP production in metabolic pathways, resulting in the generation of ROS [75]. Additionally, it has been shown that prolonged hyperglycemia and insulin resistance contribute to mitochondrial dysfunction, resulting in oxidative stress and subsequent heart injury [76]. In individuals with diabetic cardiac conditions, an excess of glucose and fatty acids has been observed to result in an elevated flow of electrons in the ETC [77]. This, in turn, leads to a partial reduction of oxygen in complex I and III, ultimately resulting in the production of superoxide (ROS). These ROS are extremely reactive and cause significant damage to not just mitochondrial DNA but also proteins and lipids, worsening mitochondrial dysfunction [78]. Therefore, this establishes a vicious cycle of mitochondrial malfunction and the production of ROS, resulting in oxidative stress and subsequent cardiac cell death.

A growing body of evidence points to oxidative stress as a contributor to the onset and progression of DCM [79]. Both in persons with diabetes and without diabetes, oxidative stress plays a critical pathophysiological role in the development of hypertrophy and remodeling, as well as in the progression of heart failure. Oxidative stress causes myofibroblasts to become fibroblasts, enhances transforming growth factor- expression, and accelerates collagen production, all of which contribute to cardiac fibrosis [80]. Several major sources of ROS production within a cardiomyocyte may lead to oxidative stress in cardiac clinical conditions. Numerous enzymes, including monoamine oxidase (MAO), xanthine oxidases (XO), and NADH oxidases (NOX), generate ROS during their catalytic activity, and alterations in the cytosolic generation of ROS contribute to the pathophysiology of myocardial dysfunction in diabetes [81, 82]. This ROS production is counterbalanced by a sophisticated anti-oxidant system that detoxifies ROS to preserve homeostasis. However, in the presence of pathological stress, increased generation of ROS and/or insufficient detoxification may cause ROS-induced damage to the DNA, nucleic acids, proteins, and lipids, resulting in cell death, which eventually results in cardiomyocyte dysfunction and death. As a result, the production, as well as detoxification of ROS must always be strictly controlled to prevent oxidative damage [64]. Research findings examining the effects of mitochondrial ROS scavenging systems on DCM in diabetic mice highlighted the causative role of mitochondrial oxidative stress by partially restoring mitochondrial function, attenuating apoptosis, enhancing cardiomyocyte contractility, and reducing ROS-induced NF-κB-mediated cardiac inflammation [41]. Administration of the mitochondria-targeted antioxidant mito-TEMPO to streptozotocin-induced diabetic db/db mice inhibited mitochondrial ROS production, prevented intra-cellular oxidative stress, reduced myocardial hypertrophy, and mitigated myocardial dysfunction in diabetic mice [83]. In STZ-induced diabetic rats, inhibition of myocardial MAO-A activity with a specific MAO-A inhibitor, clorgyline, decreased oxidative stress, apoptosis, and fibrosis, thereby restoring cardiac dysfunction [84]. Administration of apocynin, NOX inhibitor (an anti-oxidant compound) to STZ-induced diabetic mice attenuated DCM-associated injuries, improved cardiac dysfunction and elevated superoxide production, reduced cardiac hypertrophy and fibrosis, and lowered oxidative stress. It reduces the severity of DCM by suppressing apoptosis signal-regulating kinase-1 (ASK-1)-p38/JNK signaling [85]. The inhibition of XO by allopurinol in diabetic mice ameliorates type I diabetes-induced cardiac dysfunction by reducing oxidative stress and cardiac dysfunction, which may have significant clinical implications for both the treatment and prevention of DCM and vascular dysfunction [86]. Diabetes-related heart disease has been linked to endothelial nitric oxide synthase (eNOS) uncoupling, which causes an increase in the generation of ROS rather than NO. Another significant finding demonstrated that sepiapterin inhibits the uncoupling of NOS and improves LV function by increasing inducible nitric oxide synthase (iNOS) derived NO, resulting in reduced cardiac impairment in STZ-induced-diabetic mice (Fig. **1**) [87]. The aforementioned data is sufficient to support the claim that oxidative stress is a pivotal factor in the advancement of DCM. Additionally, this oxidative stress might result in the initiation of other molecular pathways that can influence the development of DCM.

## 4 MOLECULAR MECHANISMS INVOLVED IN DCMS

Studies have observed that disturbances in calcium homeostasis caused by ER stress, as well as oxidative stress resulting from mitochondrial dysfunction, might impact many signalling pathways. These pathways could potentially be targeted for the treatment and management of DCM. The following signalling pathways have been observed to have the potential to regulate the development of DCM.

### 4.1 Nucleotide-binding Oligomerization Domain-like Receptor Protein-3 (NLRP-3) Inflammasome Pathway

**Fig. (2):**
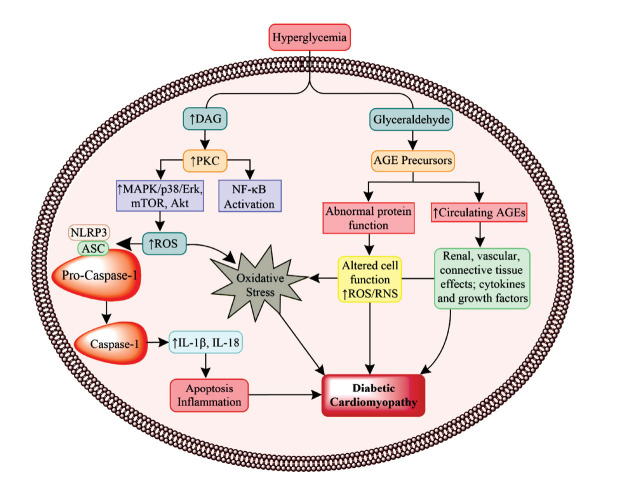
This figure depicts the various molecular signaling pathways leading to the development of diabetic cardiomyopathy.

The NLRP-3 inflammasome is a crucial component of the (innate) immune system that rapidly initiates and propagates inflammatory host defense responses against both endogenous threats and pathogenic microbial infections. Recent research has indicated that the NLRP3 inflammasome plays a role in the pathogenesis of CVDs and metabolic disorders by causing inflammation [88]. Cardiac inflammation is a prominent and early diabetic response that contributes to the emergence of DCM. The development of DCM has been linked to the NLRP-3 inflammasome. The mRNA expressions of NLRP-3, caspase-1, and IL-1 were all observed to be increased in the diabetic mice heart [89]. A multiprotein complex called the NLRP3 inflammasome is made up of three different proteins: NLRP3, pro-caspase-1, and ASC protein with a CARD domain [90]. The phenomenon can be activated by a diverse range of ligands derived from pathogens and hosts. The constituents of interest include glucose, bacterial pore-forming toxins, UV light, asbestos, hemozoin, silica, ATP, calcium pyrophosphate dihydrate, amyloid, hyaluronan, alum and environmental stimuli [91]. The triggering of the NLRP-3 inflammasome necessitates a dual process that includes priming and assembly. In the priming phase IL-1β and TNF-α, are recognized by their corresponding receptors, which include pattern recognition receptors, IL-1 receptors, and TNF receptors, resulting in the upregulation of NF-κB transcription and increased synthesis of NLRP3, pro-IL-1β, and pro-IL-18. During the assembly phase that follows, the NLRP-3 protein that has been stimulated can recognize a diverse range of stimuli that may appear unrelated. These stimuli involve particles, pathogen-associated RNA, endotoxin, and ATP. This recognition triggers the assembly of a complex macromolecular multiprotein structure consisting of NLRP-3, ASC, and pro-caspase-1, which becomes induced [92]. This multiprotein complex results in the autocleavage of pro-caspase-1 into active caspase-1, and, eventually, the cleavage and maturation of pro-IL-18 and pro-IL-1β in mature IL-18 and IL-1β, resulting in intensified inflammation and tissue damage [89]. Upon activation, caspase-1 enzymatically cleaves gasdermin D (GSDMD), resulting in the release of its active N-terminal protein, which subsequently facilitates the process of pyroptosis. Pyroptosis is a form of killed cells that is associated with inflammation and is known to have a significant impact on the progression of DCM [93]. IL-1β and -18 are produced in excess as a result of dysregulated NLRP3 inflammasome activation, which ultimately leads to pyroptotic death of inflammatory cells. Hyperglycemia-induced ROS generation increases the level of thioredoxin-interacting protein (TXNIP), which directly binds with NLRP-3 and activates it [94]. Hence, given the crucial function of the NLRP3 inflammasome in DCM, it may be a viable therapeutic approach to mitigate the likelihood of heart failure in diabetic patients by targeting the NLRP-3 inflammasome pathway [89]. The suppression of the NLRP-3 gene in rats with type 2 diabetes resulted in the mitigation of DCM by improving cardiac function and reducing cardiac inflammation, cardiomyocyte pyroptosis, and left ventricular fibrosis [94]. Metformin ameliorates DCM by inhibiting the activation of NLRP3 inflammasome, as evidenced by decreased expression of caspase-1, NLRP3, and GSDMD-NT and the substance in challenging exhibits anti-inflammatory characteristics, potentially through the inhibition of NF-κB. This inhibition may subsequently impede the activation of the NLRP3 inflammasome and/or decrease the expression of NLRP3 inflammasome components [95]. Administration of anti-aging protein Klotho to STZ-induced diabetic mice ameliorates DCM by improving cardiomyocyte apoptosis, cardiac fibrosis, and cardiac dysfunction, as well as suppressing TXNIP expression. The *in vivo* production of inflammatory mediators such as TNF-α, IL-1β, and IL-18 is associated with the induction of NLRP3 inflammasome [96]. Oral administration of Rosuvastatin, a widely used lipid-lowering drug to STZ-induced diabetic rats, alleviated cardiac dysfunction, and the suppression of inflammasome activation of NLRP3 has been found to strengthen cardiac function, reduce interstitial fibrosis and mitigate cardiac structural disorders in individuals with DCM [97]. In STZ-induced diabetic rats, administration of the traditional remedy and food mushroom *Coriolus versicolo*r resulted in protective effects against DCM *via* inhibition of TGF-1/Smad signaling and reduction of NLRP3 inflammasome activation [98]. The findings of the study indicated that Krill oils have the potential to enhance the levels of Peroxisome Proliferator-Activated Receptor-γ, Coactivator-1α, and Sirtuin-3, which have been reported to block NLRP3. This suggests that Krill oils may have a preventive effect on the pathological injuries associated with DCM [99]. Gypenosides extracted from *Gynostemma pentaphylla* (Thunb.) Makino, a traditional Chinese medicine, the study observed a reduction in C-reactive proteins (CRPs), IL-1β, and -18 levels, as well as a suppression of NLRP-3 inflammasome stimulation, resulting in the improvement of myocardial damage (Fig. **2**) [100].

### 4.2 MAPK Pathway

The MAPK is a family of highly conserved eukaryotic serine/threonine protein kinases that play a significant role in signal transduction pathways, which modulate both normal and pathophysiological cellular responses [101, 102]. Cell proliferation, differentiation, development, inflammatory reactions, and cell death are some of the physiological effects triggered in mammalian cells by signals sent by MAPK pathways [103]. The pathogenesis of DCM and heart failure is also thought to be influenced by MAPK activation. The regulation of cardiac remodeling, myocardial dysfunction, cardiac hypertrophy, fibrosis, and heart failure is governed by three noteworthy subfamilies of MAPKs, namely extra-cellular signal regulated-kinase 1/2 (ERK-1/2), p38 (MAPK), and JNK [104]. ERK1/2, a member of the MAPK family, is recognized as a contributor to cardiac hypertrophy. Several recent studies have demonstrated the crucial role of the ERK-1/2 pathway in the swift progression of DCM. Cardiac hypertrophy is a common occurrence in the advanced stages of diabetes, leading to cardiac remodeling, cardiac dysfunction, and ultimately heart failure. It is believed that the ERK-1/2 signaling cascade is responsible for the development of cardiac hypertrophy induced by high glucose levels. As a result, a diverse range of pharmaceuticals have been developed with the aim of inhibiting the ERK1/2 pathway, which may lead to a decrease in cardiac proliferation and progress. Moreover, after treatment with the ERK1/2 inhibitor PD98059, the hypertrophic responses of all cardiac cells were reduced. As a result, numerous compounds, such as PD98059 and U0126, are being developed that hinder the ERK1/2 pathway and, therefore, have the potential to inhibit cardiac growth and proliferation [105]. Administration of mito-TEMPO in diabetic mice mitigated myocardial dysfunction *via* the downregulation of ERK 1/2 phosphorylation [83]. Another type of MAPK is p38. MAPK has been found to play a crucial role in the pathophysiology of heart failure and DCM [106]. The p38 pathway has been observed to control genes that are involved in the regulation of myocyte apoptosis, cellular hypertrophy, cardiac fibrosis, and cardiac cytokine-mediated inflammation [107]. Diabetes-induced ROS can stimulate p38 MAPK, resulting in either inhibition or stimulation of cardiomyocyte apoptosis, depending on the type of p38 isoform stimulated. For instance, stimulation of p38α promotes cardiomyocyte apoptosis, whereas stimulation of p38β prevents cardiomyocyte apoptosis. Activation of p38 MAPK leads to myocyte cell hypertrophy, cardiac fibrosis, and cardiac dysfunction. Moreover, according to several studies, p38 activity suppression in the inhibition of DCM growth can be significantly achieved through the therapeutic use of a transgenic animal model or through the administration of its pharmacologic inhibitor [108]. The administration of SB203580, a pharmacological inhibitor of p38, to mice with STZ-induced DCM resulted in a substantial enhancement in cardiac function, as evidenced by a decrease in myocardial pro-inflammatory cytokine levels [107]. Oral administration of Atorvastatin, a lipophilic statin, to STZ-induced DCM Sprague-Dawley rats, decreased intramyocardial inflammation, and myocardial fibrosis, and inhibited p38 phosphorylation, resulting in improved cardiac function [109]. Intraperitoneal administration of nesfatin-1, a novel anorexigenic peptide, exerted a cardioprotective effect in STZ-induced DCM C57BL/6 J mice model by suppressing the activation of cardiac p38 and subsequently increased glucagon-like peptide-1 (GLP-1) level thereby ameliorating myocardial hypertrophy and heart dysfunction [110]. Inflammatory cytokines, sphingolipid metabolites, and oxidative stress can all stimulate JNK. Furthermore, increased JNK signaling in the hyperglycaemic heart contributes to interstitial fibrosis, endoplasmic reticulum stress, and oxidative stress [32]. The present study investigates the effects of administering an unconventional curcumin analog, namely compound (2E,6E)-2,6-bis(2-(trifluoromethyl) benzylidene), *via* oral route. The immediate inhibition of JNK kinase activity by cyclohexanone in STZ-induced C57BL/6 diabetic mice has been found to avoid inflammation and cell apoptosis caused by higher glucose, as well as DCM [111]. Treatment of STZ and high fat-fed DCM mice model with an anti-oxidant compound, apocynin extracted from *Picrorhiza kurroa* ameliorated DCM by preventing cardiomyocyte apoptosis *via* suppressing ASK-1-p38/JNK signaling cascade [85]. In a type 2 diabetes rat model, rosuvastatin alleviates DCM through the MAPK pathway’s inhibition [97]. Therefore, the inhibition of different cascades involved in MAPK signaling can serve as an excellent therapeutic tool in the management of DCM and other diabetes-associated CVDs (Fig. **2**).

### 4.3 Activation of Protein Kinase C (PKC) Pathway

The PKC has been found to have diverse functions in cardiac growth and the etiology of various cardiovascular diseases [112]. The stimulation of PKC may lead to cellular and functional changes that lead to the onset and progression of DCM and heart failure. The activation of PKC signaling in the cardiac tissue is observed as an outcome of hyperglycemia and elevated levels of growth factors, such as Ang-2, in the context of diabetes [55]. PKCs can be divided into 3 categories: conventional PKCs (which include PKCα, PKCβ, and PKCɣ); novel PKCs (which contain PKCδ, PKCε, PKCη, PKCθ); and atypical PKCs (consisting of PKCζ and PKCλ/ɩ isoforms). PKCα is the most frequently expressed PKC in cardiomyocytes. However, it has also been demonstrated that other PKC isozymes, such as PKCβ, and PKCδ, and the expression of PKCε is comparatively reduced in normal cardiac tissue, whereas it is stimulated during instances of pathological cardiac remodeling [112,113]. Conventional PKC isoforms are stimulated by calcium, phosphatidylserine (PS), and di-acylglycerol (DAG) or phorbol esters including phorbol 12-myristate 13-acetate (PMA), while the novel PKC isoforms are activated by PS, DAG as well as PMA, but not by the calcium because of the absence of a functional group at the C2 region which mediates calcium binding. Calcium, DAG, and PMA have no effect on the atypical PKCs. These atypical PKC isozymes are targets of lipid-derived secondary messengers and may be activated by lipids such as phosphatidylinositol 3,4,5-triphosphate and arachidonic acid [114]. The DAG molecule, which serves as a crucial cofactor in the activation of PKC isoforms, experiences a chronic elevation in the presence of hyperglycemia and diabetes, owing to an upsurge in the glycolytic intermediate di-hydroxy-acetone phosphate. The intermediate undergoes reduction to glycerol-3-phosphate, subsequently leading to an elevation in de novo diacylglycerol synthesis [115]. The induction of PKC is linked to various subsequent proteins and alterations in gene expression that are involved in the unique characteristics of the diabetic heart. These traits encompass cardiac fibrosis, hypertrophy, inflammation, and oxidative stress. Specifically, cardiac fibrosis is linked with plasminogen activator inhibitor-1 and TGF-β, hypertrophy is stimulated *via* MAPKs, inflammation involves NFκB and TNF-α, and oxidative stress is activated by NADPH oxidase [55]. The activation of PKC isoforms in grown vascular cells is mainly caused by hyperglycemia, specifically the β and δ isoforms. The present study reveals that in the retina of individuals with diabetes, PKC and p38α MAPK are continually activated by hyperglycemia, leading to an upregulation of Src homology-2 domain-containing phosphatase-1 (SHP-1), a protein tyrosine phosphatase that was once unidentified as a target of PKC signaling. The present cascade of signaling events culminates in the dephosphorylation of the receptor for PDGF subtype β and consequent attenuation of pathways downstream of this receptor, ultimately leading to apoptosis of pericytes. The identical pathway, which is stimulated by heightened fatty acid oxidation in arterial endothelial cells and the heart of individuals with insulin resistance, may have an equally significant impact on the development of atherosclerosis and diabetic cardiomyopathy (Giacco & Brownlee, 2010) [116]. In the cardiomyocytes of diabetic rodents and heart failure patients, the activity of the PKCβ2 isoform is elevated. Cardiomyopathy and cardiac fibrosis are manifested in transgenic mice overexpressing PKCβ2 in the myocardium, and the outcome is characterized by notable necrosis of cardiomyocytes, fibrosis, hypertrophy of the left ventricle, and compromised cardiac function [117]. In streptozotocin-induced diabetic rats, treatment with LY333531 for four weeks managed to prevent excessive PKCβ2 stimulation in the heart and reduced cardiac diastolic dysfunction [118]. Inhibition of PKCβ by Ruboxistaurin results in the attenuation of myocyte hypertrophy, collagen deposition, and diastolic dysfunction while maintaining cardiac contractility [119]. Curcumin is a naturally occurring compound that is administered to streptozotocin-induced diabetic Sprague-Dawley rats, which inhibits the translocation of PKCα and PKCβ2 to the membrane fraction, thereby preventing DCM (Fig. **2**) [120].

### 4.4 Nuclear Factor Kappa-B (NF-κB)

The NF-κB is a transcription factor implicated in the pathophysiology of numerous cardiovascular diseases [121]. A variety of physiological and non-physiological stimuli, including but not limited to cytokines, mitogens, viruses, as well as mechanical and oxidative stress, can activate NF-κB [122-124]. The activation of NF-κB in DCM can be triggered by elevated levels of circulatory glucose and LDL/VLDL lipoproteins [125]. Furthermore, they elicit the secretion of growth factors, such as CTGF and TGF-β, as well as vasoactive peptides, including Endothelin-1, Angiotensin-II, and Phenylephrine, from both circulatory and local cells [126]. The aforementioned molecules have the potential to activate NF-κB activity, either through direct or indirect means, which may be facilitated by the generation of cytokines [27]. Under strong stimuli such as cytokines or lipopolysaccharides, the degradation of IκBα occurs quickly in a matter of minutes in the canonical pathway [127]. The activation of NF-κB is primarily induced by TNFα and IL-1β through their interaction with type-1 receptors, which subsequently adhere to connector molecules such as TNF Receptor-Associated Factors [128]. After that, these adapters can interact with MAPK, which transphosphorylates and activates IKK [129]. In the non-canonical pathway, NF-κB is stimulated by cellular receptors including CD40 and lymphotoxin-βR [130]. The expression of NF-κB can be initiated directly by ROS, AGEs, and a triggered cardiac tissue RAAS in individuals with diabetes. The aforementioned phenomenon fosters maladaptive immune responses and the secretion of pro-inflammatory cytokines, including but not limited to TNF-α, MCP-1, IL-6, and IL8 [131]. The activation of NF-κB is responsible for regulating the production of genes that promote fibrosis, cytokines that cause inflammation, and cell viability, ultimately leading to impaired mitochondrial and cardiac function in individuals with diabetes. Research has demonstrated that the presence of activated NF-κB in the hearts of mice with diabetes is linked to elevated levels of ROS, superoxide, and peroxynitrite through NADPH oxidase. These events result in a decrease in the bioavailability of nitric oxide, which is necessary for vasodilation and G-protein stimulation, thus contributing to endothelial dysfunction. Peroxynitrite can oxidize the sulfhydryl groups in enzymes needed for the mitochondrial ETC. Additionally, peroxynitrite is believed to contribute to cardiac contractile dysfunction by inhibiting creatine kinase activity in myofibrils. Given the crucial involvement of NF-κB in the pathophysiological mechanisms underlying various aspects of DCM, it is reasonable to hypothesize that external manipulation of NF-κB activation could prove efficacious in the development of novel therapeutic interventions. The study demonstrated that the use of pyrrolidine dithiocarbamate to inhibit NF-κB resulted in improved mitochondrial structural integrity and reduced oxidative stress. This led to an increase in ATP synthesis and NO bioavailability, ultimately resulting in the restoration of cardiac function in individuals with type 2 diabetes [132]. Administration of resveratrol, a phenolic antioxidant in fructose-induced diabetic rats, activates SIRT-1, which deacetylates NF-κB and reduces the transcription of NOX subunits, thereby attenuating oxidative stress and cardiac hypertrophy [121]. The present study investigated the effects of apigenin, a flavonoid compound, on the occurrence and advancement of DCM in male C57/BL6J mice induced with STZ. The results indicate that apigenin treatment reduces DCM by blocking the translocation of NF-κB, decreasing the amount of MDA and TNF-α, and boosting the levels of SOD and GPx in the left ventricular tissue [133]. Administration of kaempferol, a flavonoid, has the potential to attenuate DCM in STZ-induced diabetic rats through the upregulation of SIRT1 and a decrease in the activation of NF-κB (Fig. **2**) [134].

## 5 DIAGNOSTIC TECHNIQUES

The majority of DCM instances are subclinical, so individuals can fail to demonstrate noticeable signs or disease-related indicators. The cardiomyocytes only experience substructural changes in the early phases, and only extremely sensitive techniques, such as strain, strain rate, and myocardial tissue velocity, can detect them. In the middle stage of DCM, myocyte hypertrophy and fibrosis develop, which may be related to structural alterations such as LV hypertrophy and increased muscle mass. Conventional diagnostic methods, such as echocardiography, may detect diastolic or systolic dysfunction at this point. Yet, DCM tends to be only discovered when HF has begun to develop, and systolic dysfunction has been identified as an aftereffect of the pathology. Non-invasive tests are performed on diabetic patients who present to emergency rooms with symptoms of HF. These tests include chest X-rays to detect fluid buildup in the lungs, electrocardiograms to detect ventricular overload, and conventional cardiac ultrasounds to detect structural and functional abnormalities of the myocardium [26]. An accurate method to diagnose DCM could include endomyocardial biopsy sampling and cardiac catheterization. Nevertheless, this particular approach is intrusive and lacks specificity in terms of identifying diastolic dysfunction linked to DCM. The utilization of the ratio between early passive transmitral inflow velocity (E) and the velocity of the medial mitral annulus (e) serves as a substitute for extensively measuring left ventricular filling pressure and is a dependable prognostic marker for individuals diagnosed with diabetes [135]. The presence of anomalies in E/e' has been linked to the onset of cardiac failure and heightened rates of fatality [136]. Non-invasive methods are the preferred approach for achieving this objective. Over the past 20 years, non-invasive imaging methods that provide accurate information about the morphological features and functions of the heart, such as nuclear imaging, echocardiography, magnetic resonance imaging (MRI), and electrocardiography, have greatly evolved [136]. In diabetic patients, a broad spectrum of cardiovascular plasma/ serum biomarkers has been described. These biomarkers include matrix metalloproteinases (MMPs), cardiac troponins, and brain natriuretic peptides (BNP). Changes in the concentrations of these biomarkers may indicate both structural and functional myocardial dysfunction [137]. However, there are several limitations associated with serum biomarkers in clinical practice. Most serum bio-markers, including BNP and troponins, are not specific to DCM. Their levels are elevated in several heart diseases, such as heart failure, myocardial infarction, and hypertension. This lack of specificity makes it difficult to differentiate between DCM and other types of heart dysfunction [138]. The sensitivity of serum bio-markers varies according to the stage and severity of DCM. Early-stage DCM may not produce significantly raised levels of bio-marker, resulting to delayed diagnosis. As a result, relying simply on these bio-markers may not capture the entire scope of the disease, especially in slightly symptomatic individuals [139]. Diabetic patients may have many comorbidities, including chronic kidney disease (CKD) and liver disease, which might influence the levels of bio-markers. For example, regardless of cardiac state, CKD might result in increased BNP levels. This complicating factor might lead to misunderstanding of results in DCM individuals with co-existing diseases [140]. While bio-markers (BNP) have predictive significance for unfavourable cardiovascular outcomes, their ability to predict development in DCM is limited [140]. The relationship between bio-marker levels and the progression of structural and functional cardiac abnormalities in diabetes is not fully understood. Combining bio-markers with imaging techniques and clinical assessment remains crucial for a comprehensive evaluation of DCM. The main imaging approaches or diagnostic tests for DCM identification are given in Table **1**. Additionally, more advanced techniques such as Speckle tracking echocardiography (STE) and fluydodynamic study can also be employed. STE is a more sophisticated type of echocardiography that tracks the movements of natural acoustic markers (speckles) inside the heart to measure cardiac function in greater detail [141, 142]. It gives more information than a standard echocardiogram. Fluid dynamics studies examine blood flow and pressures in the cardiovascular system. These investigations can be conducted utilising a variety of methodologies, including MRI with flow mapping and computational fluid dynamics [143].

## 6 THERAPEUTIC INTERVENTIONS INVOLVED IN THE TREATMENT OF DCM

Changing one's lifestyle, regulating blood glucose levels, modification of risk factors for cardiovascular disease, lipid-lowering therapy, and management of heart failure comprise the cornerstones of DCM treatment (Table **2**) [11]. The implementation of weight loss, consistent physical activity, and caloric limitation can have a beneficial impact on metabolic irregularities and improve insulin resistance by enhancing post-receptor insulin signaling and insulin-mediated glucose transport [131]. In numerous clinical studies, physical activity was linked to a significant decrease in CVD and mortality in people with diabetes [150]. Both animal studies and human subjects have demonstrated that exercise training improves myocardial activity, glycemic control, and cardiorespiratory fitness,, thereby decreasing in the incidence of DCM [151]. Regulating blood glucose levels is a crucial objective in managing diabetic cardiomyopathy. Diabetes triggers the activation and up-regulation of the RAAS. Therefore, inhibiting RAAS with angiotensin receptor blockers (ARB) and ACE inhibitors may reverse and halt the progression of DCM [152]. The utilization of beta-blockers, calcium channel blockers, and hypolipidemic drugs has been shown to be efficacious in both primary and secondary prevention of cardiovascular events.

## 7 CLINICAL PROSPECTS

As there is still a need to explore more and more agents that help in the treatment of DCM. To progress the research and investigate, extensive and targeted experiments are required. Therefore, Table **3** consists of all studies that are exploring the protective effects of various agents for the treatment of DCM.

## CONCLUSION

The cause of DCM is complex, comprising a combination of several variables which have important consequences in clinical practice. Our review explores several pathogenic pathways, including hyperinsulinemia, insulin resistance, hyperglycemia, and chronic hyperglycemia. These mechanisms all contribute to endoplasmic reticulum stress, calcium dyshomeostasis, mitochondrial dysfunction, and oxidative stress. Moreover, the stimulation of the renin-angiotensin-aldosterone system (RAAS) and other pathogenic variables are significant contributors to the advancement and formation of DCM. We have also investigated the participation of signalling pathways, such as NLRP3, MAPK, PKC, and NF-κB, as well as the existing diagnostic tools and therapeutic treatments for the management of DCM.

In order to gain a deeper understanding of the intricate mechanisms involved in DCM, it is crucial for future studies to prioritize exploring the precise molecular pathways that contribute to endoplasmic reticulum stress and mitochondrial dysfunction. It is important to focus on comprehending the significance of NLRP3 inflammasome activation and how it interacts with insulin resistance and oxidative stress. In addition, investigating the interaction between the RAAS and other signalling pathways, such as MAPK and PKC, could offer a more profound understanding of possible targets for therapy. There should be an expansion of clinical trials that focus on these pathways and use new therapies to confirm their effectiveness in improving patient outcomes. By dedicating our investigation efforts to this specific area, we can enhance our comprehension of DCM and devise more efficient approaches to prevent and cure it.

## Figures and Tables

**Table 1:** The various imaging approaches or diagnostic tests for DCM.

**S. No.**	**Diagnostic Tests**		**Principle**	**Findings**	**References**
1.	Echocardiography	Conventional Echocardiography	Its utilization *via* Doppler technology enables the assessment of aberrant flows.	Diastolic and systolic dysfunction, Left ventricular hypertrophy, Epicardial adipose tissue (EAT) accumulation.	[144, 145]
		Tissue Doppler echocardiography	The quantification of velocities through myocardial tissue.	Diastolic dysfunction, impairment in radial direction while analyzing strain rate and global and regional ventricular strains.	
		Intravenous contrast echocardiography	The enhanced reflectivity of intravenous contrast agents (micro-bubbles) is attributed to the distinct reflection of the enclosed gas in comparison to the adjacent blood and tissue.	Altered contractility and systolic function.	
		3D Echocardiography	The utilization of 3D-Echo enables the acquisition of a comprehensive pyramidal dataset in actual time, thereby facilitating the assessment of ventricles that exhibit anomalous shapes or mobility.	Left ventricular diastolic and systolic dysfunction, abnormal wall motions in ventricles.	[26, 141]
		Speckle tracking echocardiography (STE)	Analysis of tissue deformation and motion by speckle using 2D/3D echo, 2D/3D interference patterns, and acoustic reflections.	Alteration in myocardial deformation, decreased circumferential and Longitudinal systolic strain, right systolic and left ventricular dysfunction.	
2.	Electrocardiography		Records electrical signals in the heart to determine heart activity.	Prolonged P wave, QRS and QT intervals, Elevated ST segment, ventricular overload.	[146]
3.	Magnetic Resonance Imaging (MRI)	Phase MRI	Utilizes variations in signal intensity through valves to detect changes in blood flow.	The individual exhibits compromised left ventricular function hindered cardiac metabolic processes, and a decreased ratio of myocardial phosphocreatine to adenosine-triphosphate.	[26, 143, 147]
		Gradient MRI	The present study investigates the utilization of radiofrequency pulses in conjunction with an electrocardiogram for the cine display of the cardiac cycle.	Impact on right ventricle dimensions and function.	
		Tagged MRI	Radiofrequency pulse-induced cardiac distortion.	The present study examines the regional contractility of the left ventricular in the context of systolic dysfunction, as well as the impact of poor glycemic control on decreased circumferential and longitudinal strains and strain rates.	
4.	Nuclear imaging	SPECT (single photon emission CT)	Image-coupled electrocardiogram acquisition is guided by a gamma-ray radioisotope.	Perfusion and functional alterations.	[148]
		Positron emission tomography (PET)	Indirect detection of gamma-ray pairs is possible by a radionuclide that emits positrons and generates 3D pictures using CT analysis.	Perfusion alterations.	
5.	Cardiac Computed Tomography (CCT)		Cross-sectional tomography uses computer-processed combinations of x-ray images.	Myocardial ischemia calcification left ventricular dysfunction.	[145]
6.	Serum biomarkers	Matrix metalloproteinases (MMPs)	Laboratory analysis to measure the amount of serum biomarkers released in the blood.	Elevated matrix turnover, elevated concentrations of MMPs, specifically MMP9, and reduced concentrations of tissue inhibitors of MMPs.	[137, 147, 149]
		Cardiac troponins		Increased troponin T levels	
		Brain natriuretic peptide (BNP)		Increased level of BNP indicates left ventricular systolic dysfunction.	
		Serum aminoterminal propeptide type I and type III PIIINP		Increased level of PIIINP indicates LV dysfunction.	

**Table 2:** This table summarizes the various pharmacological treatments for DCM.

**S. No.**	**Treatment Type**		**Mechanism of Action**	**Description**	**Side Effects**	**Contraindications**	**References**
1.	Glycaemic control	Metformin, sulfonylureas, pioglitazones	↓Glucose production, ↑Insulin sensitivity, ↓Insulin resistance, ↓TNF-α activity, ↑endothelial NO production, ↓ROS generation, ↓collagen production, ↓p53 expression, ↓ cardiomyocytes, and fibroblasts left ventricle remodeling, Improves systolic and diastolic parameters. Stimulates prokineticin 2 pathway→↑Cardiac function, ↓Apoptosis ↑Cardiomyocyte autophagy	Maintains normal blood glucose level and reduces the risk of developing DCM	Weight gain, cardiovascular risk, gastrointestinal issues, vitamin-B12 deficiency	Pregnancy, breast feeding, hypersensitivity to drug	[153]
2.	𝛽-Blockers	Carvedilol, Metoprolol	Antagonizes both α and β receptors	The prevention and reversal of cardiac remodeling leads to enhanced left ventricular function	Fatigue, bradycardia, dizziness, weight gain, sexual dysfunction, mood changes	Cardiogenic shock, asthma, depression, hypersensitivity	[154, 155]
3.	ACE inhibitors	Ramipril	This statement suggests that ACE inhibition leads to a reduction in plasma BNP levels and a rise in glucose uptake in skeletal muscle through GLUT-4 translocation	lessen cardiac hypertrophy and enhance LV function	Dry cough, hyperkalemia, dizziness, fatigue, altered taste, kidney injury	Pregnancy, severe hyperkalemia, and hypotension, hypersensitivity	[152, 156]
4.	Angiotensin II receptor antagonists	Candesartan, Telmisartan	Blocks effects of angiotensin II	Attenuates myocardial fibrosis by promoting collagen degradation in patients with diabetes	Dizziness, headache, fatigue, gastrointestinal symptoms	Pregnancy, hypersensitivity, hypotension	[137, 157]
5.	Calcium channel antagonists	Diltiazem	During depolarization, it inhibits the inflow of Ca^2+^ ions into the cardiac muscle	Suppress degeneration, hypertrophy, atrial vasodilation, and fibrosis caused by hyperglycemia	Bradycardia, constipation, headache, flushing, fatigue, dizziness	Hypotension, sick sinus syndrome, hypersensitivity	[158]
		Verapamil	It acts by preventing the expression of the pro-apoptotic TXNIP, which prevents apoptosis of β-cells and increases endogenous insulin levels, improving survival and functioning of β-cell survival	Enhances vasodilation and left ventricular relaxation, with a reduction in LV systolic and diastolic dysfunction	Constipation, bradycardia, dizziness, fatigue, headache	Hypotension, sick sinus syndrome, hypersensitivity	[159]
6.	Lipid-lowering drugs	Simvastatin	Inhibits hyperglycemia-induced cardiomyocyte apoptosis by decreasing inflammation and ROS production	Reduce collagen production and fibrosis	Muscle pain, nausea, headache, constipation, hepatotoxicity	Pregnancy, lactation, hypersensitivity, liver disease	[160, 161]
-	-	Atorvastatin	Decreases β-adrenergic dysfunction and restores the positive inotropic effect of β-adrenoceptor stimulation through an increase in NO production from endothelial cells *via* neuronal NO synthase	Improves functioning of LV by reducing myocardial fibrosis and intramyocardial inflammation	Muscle pain, nausea, headache, constipation, hepatotoxicity, Myopathy, hepatotoxicity, cognitive effects	Pregnancy, lactation, hypersensitivity, liver disease	[162]
		The combined therapy of atorvastatin and metformin	Activates AMPK/SIRT1 signaling pathway	The study observed a notable reduction in oxidative stress and a rise in the levels of inflammation-associated proteins such as caspase-1, NLRP-3, and IL-1β, P-p65/p65, and TLR-4, reduced expression of pro-apoptotic-related proteins (Bax & caspase-3)	Liver and renal dysfunction	Pregnancy, lactation, hypersensitivity, liver disease	[163]
		Rosuvastatin	This statement suggests that the activation of NLRP-3 inflammasome is inhibited through the suppression of TXNIP and MAPK pathway	Protect cardiac function	Muscle pain, nausea, headache, constipation, hepatotoxicity	Pregnancy, lactation, hypersensitivity, liver disease	[97]
7.	TZDs	Pioglitazone, Rosiglitazone	Act by increasing insulin sensitivity in skeletal muscle through binding and activation of PPAR-𝛿 and also act on PPAR-⍺ and increase serum HDL cholesterol, decrease serum triacylglycerols and increase LDL cholesterol levels	Improves glucose metabolism and reduces NEFA (non-esterified fatty acids) utilization by the myocardium	Weight gain, headache, fatigue, muscle pain, hepatotoxicity	Pregnancy, liver diseases, history of heart failure	[164]

**Table 3:** Ongoing clinical trials in diabetic cardiomyopathy.

**Study Title**	**Intervention/Diagnostic Test**	**NCT Number**
Evaluation of alpha lipoic acid in diabetic cardiomyopathy	Physiomance acide lipoïque gold	NCT04141475
Alpha lipoic acid in diabetic patients with ischaemic cardiomyopathy	Alpha lipoic acid 600 MG oral tablet	NCT06056687
AT-001 safety and efficacy in diabetic cardiomyopathy patients	AT-001	NCT04083339
The efficacy of trimetazidine in diabetic patients	Trimetazidine dihydrochloride	NCT05556005
The Impact of high-intensity interval training on cardiac function and glycaemic control in diabetic cardiomyopathy	high-intensity interval exercise training	NCT03299790
Mitochondrial function of the heart-*in vivo* and *ex vivo*	31P-MRS scan and, cardiac MRI scan and blood analysis	NCT03049228
Mitochondrial substrate utilization in the diabetic human heart	Surgery	NCT05958706

## LIST OF ABBREVIATIONS

BNP: Brain Natriuretic Peptides
DAG: Diacylglycerol
DCM: Diabetic Cardiomyopathy
DCvds: Diabetic Cardiovascular Diseases
ER: Endoplasmic Reticulum
ERAD: ER-Associated Degradation
FFA: Free Fatty Acids
IRE-1: Inositol-Requiring Enzyme-1
MCP-1: Monocyte Chemoattractant Protein-1
MMPs: Matrix Metalloproteinases
RAAS: Renin-Angiotensin-Aldosterone System
UPR: Unfolding Protein Response
WHO: World Health Organization

## References

[1] Martín-Timón I., Sevillano-Collantes C., Segura-Galindo A., Del Cañizo-Gómez F.J. (2014). Type 2 diabetes and cardiovascular disease: Have all risk factors the same strength?. World J. Diabetes.

[2] Zhou Y., Suo W., Zhang X. (2023). Targeting epigenetics in diabetic cardiomyopathy: Therapeutic potential of flavonoids.. Biomed. Pharmacother..

[3] Tang Z., Wang P., Dong C., Zhang J., Wang X., Pei H. (2022). Oxidative stress signaling mediated pathogenesis of diabetic cardiomyopathy.. Oxid. Med. Cell. Longev..

[4] Devi S., Chauhan S., Mannan A., Singh T.G. (2024). Targeting cardiovascular risk factors with eugenol: an anti-inflammatory perspective.. Inflammopharmacology.

[5] Kim A.H., Jang J.E., Han J. (2022). Current status on the therapeutic strategies for heart failure and diabetic cardiomyopathy.. Biomed. Pharmacother..

[6] Behl T., Bungau S., Kumar K. (2020). Pleotropic Effects of polyphenols in cardiovascular system.. Biomed. Pharmacother..

[7] Yang L., Zhao D., Ren J., Yang J. (2015). Endoplasmic reticulum stress and protein quality control in diabetic cardiomyopathy.. Biochim. Biophys. Acta Mol. Basis Dis..

[8] Lee Y.B., Han K., Kim B. (2019). Risk of early mortality and cardiovascular disease in type 1 diabetes: a comparison with type 2 diabetes, a nationwide study.. Cardiovasc. Diabetol..

[9] Grubić Rotkvić P., Planinić Z., Liberati Pršo A.M., Šikić J., Galić E., Rotkvić L. (2021). The mystery of diabetic cardiomyopathy: From early concepts and underlying mechanisms to novel therapeutic possibilities.. Int. J. Mol. Sci..

[10] Corb Aron R.A., Abid A., Vesa C.M. (2021). Recognizing the benefits of pre-/probiotics in metabolic syndrome and type 2 diabetes mellitus considering the influence of Akkermansia muciniphila as a key gut bacterium.. Microorganisms.

[11] Trachanas K., Sideris S., Aggeli C. (2014). Diabetic cardiomyopathy: from pathophysiology to treatment.. Hellenic J. Cardiol..

[12] Rubler S., Dlugash J., Yuceoglu Y.Z., Kumral T., Branwood A.W., Grishman A. (1972). New type of cardiomyopathy associated with diabetic glomerulosclerosis.. Am. J. Cardiol..

[13] Sears B., Perry M. (2015). The role of fatty acids in insulin resistance.. Lipids Health Dis..

[14] Goldberg I.J., Trent C.M., Schulze P.C. (2012). Lipid metabolism and toxicity in the heart.. Cell Metab..

[15] Kolczynska K., Loza-Valdes A., Hawro I., Sumara G. (2020). Diacylglycerol-evoked activation of PKC and PKD isoforms in regulation of glucose and lipid metabolism: a review.. Lipids Health Dis..

[16] Field B.C., Gordillo R., Scherer P.E. (2020). The role of ceramides in diabetes and cardiovascular disease regulation of ceramides by adipokines.. Front. Endocrinol. (Lausanne).

[17] Petersen M.C., Shulman G.I. (2018). Mechanisms of insulin action and insulin resistance.. Physiol. Rev..

[18] Kruszewska J., Cudnoch-Jedrzejewska A., Czarzasta K. (2022). Remodeling and fibrosis of the cardiac muscle in the course of obesity-pathogenesis and involvement of the extracellular matrix.. Int. J. Mol. Sci..

[19] Clemente-Suárez V.J., Redondo-Flórez L., Beltrán-Velasco A.I. (2023). The role of adipokines in health and disease.. Biomedicines.

[20] Boucher J., Kleinridders A., Kahn C.R. (2014). Insulin receptor signaling in normal and insulin-resistant states.. Cold Spring Harb. Perspect. Biol..

[21] Meex R.C.R., Blaak E.E., van Loon L.J.C. (2019). Lipotoxicity plays a key role in the development of both insulin resistance and muscle atrophy in patients with type 2 diabetes.. Obes. Rev..

[22] Dabravolski S.A., Sadykhov N.K., Kartuesov A.G., Borisov E.E., Sukhorukov V.N., Orekhov A.N. (2022). The role of mitochondrial abnormalities in diabetic cardiomyopathy.. Int. J. Mol. Sci..

[23] Bhargava S.K., Singh T.G., Mannan A., Singh S., Singh M., Gupta S. (2022). Pharmacological evaluation of Thuja occidentalis for the attenuation of neuropathy *via* AGEs and TNF-α inhibition in diabetic neuropathic rats.. Environ. Sci. Pollut. Res. Int..

[24] Bhargava S.K., Singh T.G., Mannan A., Singh S., Gupta S. (2022). Pharmacological evaluation of Thuja occidentalis for the attenuation of nephropathy in streptozotocin-induced diabetes rats.. Obes. Med..

[25] Kumar S., Behl T., Sachdeva M. (2021). Implicating the effect of ketogenic diet as a preventive measure to obesity and diabetes mellitus.. Life Sci..

[26] Lorenzo-Almorós A., Tuñón J., Orejas M., Cortés M., Egido J., Lorenzo Ó. (2017). Diagnostic approaches for diabetic cardiomyopathy.. Cardiovasc. Diabetol..

[27] Fang Z.Y., Prins J.B., Marwick T.H. (2004). Diabetic cardiomyopathy: evidence, mechanisms, and therapeutic implications.. Endocr. Rev..

[28] Chavali V., Tyagi S.C., Mishra P.K. (2013). Predictors and prevention of diabetic cardiomyopathy.. Diabetes Metab. Syndr. Obes..

[29] Falcão-Pires I., Leite-Moreira A.F. (2012). Diabetic cardiomyopathy: understanding the molecular and cellular basis to progress in diagnosis and treatment.. Heart Fail. Rev..

[30] Xu G., Chen J., Jing G., Shalev A. (2012). Preventing β-cell loss and diabetes with calcium channel blockers.. Diabetes.

[31] Meusser B., Hirsch C., Jarosch E., Sommer T. (2005). ERAD: the long road to destruction.. Nat. Cell Biol..

[32] Jia G., Whaley-Connell A., Sowers J.R. (2018). Diabetic cardiomyopathy: a hyperglycaemia- and insulin-resistance-induced heart disease.. Diabetologia.

[33] Sun S., Yang S., An N. (2019). Astragalus polysaccharides inhibits cardiomyocyte apoptosis during diabetic cardiomyopathy *via* the endoplasmic reticulum stress pathway.. J. Ethnopharmacol..

[34] Wu T., Dong Z., Geng J. (2011). Valsartan protects against ER stress-induced myocardial apoptosis *via* CHOP/Puma signaling pathway in streptozotocin-induced diabetic rats.. Eur. J. Pharm. Sci..

[35] Guo R., Liu W., Liu B., Zhang B., Li W., Xu Y. (2015). SIRT1 suppresses cardiomyocyte apoptosis in diabetic cardiomyopathy: An insight into endoplasmic reticulum stress response mechanism.. Int. J. Cardiol..

[36] Yu H., Zhen J., Yang Y., Gu J., Wu S., Liu Q. (2016). Ginsenoside Rg1 ameliorates diabetic cardiomyopathy by inhibiting endoplasmic reticulum stress-induced apoptosis in a streptozotocin-induced diabetes rat model.. J. Cell. Mol. Med..

[37] Krebs J., Agellon L.B., Michalak M. (2015). Ca2+ homeostasis and endoplasmic reticulum (ER) stress: An integrated view of calcium signaling.. Biochem. Biophys. Res. Commun..

[38] Xu J., Zhou Q., Xu W., Cai L. (2012). Endoplasmic reticulum stress and diabetic cardiomyopathy.. Exp. Diabetes Res..

[39] Matuz-Mares D., González-Andrade M., Araiza-Villanueva M.G., Vilchis-Landeros M.M., Vázquez-Meza H. (2022). Mitochondrial calcium: Effects of its imbalance in disease.. Antioxidants.

[40] Battiprolu P.K., Gillette T.G., Wang Z.V., Lavandero S., Hill J.A. (2010). Diabetic cardiomyopathy: mechanisms and therapeutic targets.. Drug Discov. Today Dis. Mech..

[41] Gollmer J., Zirlik A., Bugger H. (2019). Established and emerging mechanisms of diabetic cardiomyopathy.. J. Lipid Atheroscler..

[42] Pereira L., Matthes J., Schuster I. (2006). Mechanisms of [Ca2+]i transient decrease in cardiomyopathy of db/db type 2 diabetic mice.. Diabetes.

[43] Trost S.U., Belke D.D., Bluhm W.F., Meyer M., Swanson E., Dillmann W.H. (2002). Overexpression of the sarcoplasmic reticulum Ca(2+)-ATPase improves myocardial contractility in diabetic cardiomyopathy.. Diabetes.

[44] Al Kury L.T. (2020). Calcium homeostasis in ventricular myocytes of diabetic cardiomyopathy.. J. Diabetes Res..

[45] Yoast R.E., Emrich S.M., Zhang X. (2021). The Mitochondrial Ca2+ uniporter is a central regulator of interorganellar Ca2+ transfer and NFAT activation.. J. Biol. Chem..

[46] Lee S.H., Duron H.E., Chaudhuri D. (2023). Beyond the TCA cycle: new insights into mitochondrial calcium regulation of oxidative phosphorylation.. Biochem. Soc. Trans..

[47] Patti M.E., Corvera S. (2010). The role of mitochondria in the pathogenesis of type 2 diabetes.. Endocr. Rev..

[48] Duncan J.G. (2011). Mitochondrial dysfunction in diabetic cardiomyopathy.. Biochim. Biophys. Acta Mol. Cell Res..

[49] Garg N., Singh T.G., Khan H., Arora S., Kaur A., Mannan A. (2021). Mechanistic interventions of selected ocimum species in management of diabetes, obesity and liver disorders: Transformative Developments from Preclinical to Clinical Approaches.. Biointerface Res. Appl. Chem..

[50] Jia G., Hill M.A., Sowers J.R. (2018). Diabetic cardiomyopathy.. Circ. Res..

[51] Behera R., Sharma V., Grewal A.K. (2023). Mechanistic correlation between mitochondrial permeability transition pores and mitochondrial ATP dependent potassium channels in ischemia reperfusion.. Biomed. Pharmacother..

[52] Chen J., Zhang Z., Cai L. (2014). Diabetic cardiomyopathy and its prevention by Nrf2: current status.. Diabetes Metab. J..

[53] Cai L., Klein J.B., Kang Y.J. (2000). Metallothionein inhibits peroxynitrite-induced DNA and lipoprotein damage.. J. Biol. Chem..

[54] Fyhrquist F., Saijonmaa O. (2008). Renin-angiotensin system revisited.. J. Intern. Med..

[55] Huynh K., Bernardo B.C., McMullen J.R., Ritchie R.H. (2014). Diabetic cardiomyopathy: Mechanisms and new treatment strategies targeting antioxidant signaling pathways.. Pharmacol. Ther..

[56] Lee W.S., Kim J. (2017). Diabetic cardiomyopathy: where we are and where we are going.. Korean J. Intern. Med. (Korean. Assoc. Intern. Med.).

[57] Boudina S., Abel E.D. (2010). Diabetic cardiomyopathy, causes and effects.. Rev. Endocr. Metab. Disord..

[58] Manrique C., Lastra G., Habibi J. (2007). Methods in the evaluation of cardiovascular renin angiotensin aldosterone activation and oxidative stress.. Methods Mol. Med..

[59] Cooper S.A., Whaley-Connell A., Habibi J. (2007). Renin-angiotensin-aldosterone system and oxidative stress in cardiovascular insulin resistance.. Am. J. Physiol. Heart Circ. Physiol..

[60] Catena C., Colussi G., Brosolo G., Iogna-Prat L., Sechi L.A. (2012). Aldosterone and aldosterone antagonists in cardiac disease: what is known, what is new.. Am. J. Cardiovasc. Dis..

[61] Mandavia C.H., Aroor A.R., DeMarco V.G., Sowers J.R. (2013). Molecular and metabolic mechanisms of cardiac dysfunction in diabetes.. Life Sci..

[62] Westermann D., Rutschow S., Jäger S. (2007). Contributions of inflammation and cardiac matrix metalloproteinase activity to cardiac failure in diabetic cardiomyopathy: the role of angiotensin type 1 receptor antagonism.. Diabetes.

[63] Syed A.A., Reza M.I., Shafiq M. (2022). Cissus quadrangularis extract mitigates diabetic cardiomyopathy by inhibiting RAAS activation, inflammation and oxidative stress.. Biomarkers.

[64] Byrne N.J., Rajasekaran N.S., Abel E.D., Bugger H. (2021). Therapeutic potential of targeting oxidative stress in diabetic cardiomyopathy.. Free Radic. Biol. Med..

[65] Singh R., Farooq S.A., Mannan A. (2022). Animal models of diabetic microvascular complications: Relevance to clinical features.. Biomed. Pharmacother..

[66] Zhang X., Chen C. (2012). A new insight of mechanisms, diagnosis and treatment of diabetic cardiomyopathy.. Endocrine.

[67] Mannan A., Garg N., Singh T.G., Kang H.K. (2021). Peroxisome proliferator-activated receptor-gamma (PPAR-ɣ): Molecular effects and its importance as a novel therapeutic target for cerebral ischemic injury.. Neurochem. Res..

[68] Herrero P., Peterson L.R., McGill J.B. (2006). Increased myocardial fatty acid metabolism in patients with type 1 diabetes mellitus.. J. Am. Coll. Cardiol..

[69] Parim B., Sathibabu Uddandrao V.V., Saravanan G. (2019). Diabetic cardiomyopathy: molecular mechanisms, detrimental effects of conventional treatment, and beneficial effects of natural therapy.. Heart Fail. Rev..

[70] Arora A., Behl T., Sehgal A. (2021). Unravelling the involvement of gut microbiota in type 2 diabetes mellitus.. Life Sci..

[71] An D., Rodrigues B. (2006). Role of changes in cardiac metabolism in development of diabetic cardiomyopathy.. Am. J. Physiol. Heart Circ. Physiol..

[72] Velez M., Kohli S., Sabbah H.N. (2014). Animal models of insulin resistance and heart failure.. Heart Fail. Rev..

[73] Lee T.I., Kao Y.H., Chen Y.C., Huang J.H., Hsiao F.C., Chen Y.J. (2013). Peroxisome proliferator-activated receptors modulate cardiac dysfunction in diabetic cardiomyopathy.. Diabetes Res. Clin. Pract..

[74] Bugger H., Abel E.D. (2014). Molecular mechanisms of diabetic cardiomyopathy.. Diabetologia.

[75] Baev A.Y., Vinokurov A.Y., Novikova I.N., Dremin V.V., Potapova E.V., Abramov A.Y. (2022). Interaction of mitochondrial calcium and ROS in neurodegeneration.. Cells.

[76] Bhatti J.S., Bhatti G.K., Reddy P.H. (2017). Mitochondrial dysfunction and oxidative stress in metabolic disorders - A step towards mitochondria based therapeutic strategies. Biochimica et Biophysica Acta (BBA) -.. Mol Basis Dis.

[77] Nishikawa T., Edelstein D., Du X.L. (2000). Normalizing mitochondrial superoxide production blocks three pathways of hyperglycaemic damage.. Nature.

[78] Zorov D.B., Juhaszova M., Sollott S.J. (2014). Mitochondrial reactive oxygen species (ROS) and ROS-induced ROS release.. Physiol. Rev..

[79] Tarquini R., Lazzeri C., Pala L., Rotella C.M., Gensini G.F. (2011). The diabetic cardiomyopathy.. Acta Diabetol..

[80] Zhao W., Zhao T., Chen Y., Ahokas R.A., Sun Y. (2008). Oxidative stress mediates cardiac fibrosis by enhancing transforming growth factor-beta1 in hypertensive rats.. Mol. Cell. Biochem..

[81] Kaludercic N., Mialet-Perez J., Paolocci N., Parini A., Di Lisa F. (2014). Monoamine oxidases as sources of oxidants in the heart.. J. Mol. Cell. Cardiol..

[82] Mannan A., Singh T.G., Singh V., Garg N., Kaur A., Singh M. (2022). Insights into the mechanism of the therapeutic potential of herbal monoamine oxidase inhibitors in neurological diseases.. Curr. Drug Targets.

[83] Ni R., Cao T., Xiong S. (2016). Therapeutic inhibition of mitochondrial reactive oxygen species with mito-TEMPO reduces diabetic cardiomyopathy.. Free Radic. Biol. Med..

[84] Umbarkar P., Singh S., Arkat S., Bodhankar S.L., Lohidasan S., Sitasawad S.L. (2015). Monoamine oxidase-A is an important source of oxidative stress and promotes cardiac dysfunction, apoptosis, and fibrosis in diabetic cardiomyopathy.. Free Radic. Biol. Med..

[85] Ding W., Feng H., Li W.J. (2021). Apocynin attenuates diabetic cardiomyopathy by suppressing ASK1-p38/JNK signaling.. Eur. J. Pharmacol..

[86] Rajesh M., Mukhopadhyay P., Bátkai S. (2009). Xanthine oxidase inhibitor allopurinol attenuates the development of diabetic cardiomyopathy.. J. Cell. Mol. Med..

[87] Jo H., Otani H., Jo F. (2011). Inhibition of nitric oxide synthase uncoupling by sepiapterin improves left ventricular function in streptozotocin-induced diabetic mice.. Clin. Exp. Pharmacol. Physiol..

[88] Toldo S., Mezzaroma E., Buckley L.F. (2022). Targeting the NLRP3 inflammasome in cardiovascular diseases.. Pharmacol. Ther..

[89] Sun Y., Ding S. (2021). NLRP3 Inflammasome in diabetic cardiomyopathy and exercise intervention.. Int. J. Mol. Sci..

[90] Swanson K.V., Deng M., Ting J.P.Y. (2019). The NLRP3 inflammasome: molecular activation and regulation to therapeutics.. Nat. Rev. Immunol..

[91] Luo B., Huang F., Liu Y. (2017). NLRP3 Inflammasome as a molecular marker in diabetic cardiomyopathy.. Front. Physiol..

[92] Ding K., Song C., Hu H., Yin K., Huang H., Tang H. (2022). The role of NLRP3 inflammasome in diabetic cardiomyopathy and its therapeutic implications.. Oxid. Med. Cell. Longev..

[93] Robinson N., Ganesan R., Hegedűs C., Kovács K., Kufer T.A., Virág L. (2019). Programmed necrotic cell death of macrophages: Focus on pyroptosis, necroptosis, and parthanatos.. Redox Biol..

[94] Luo B., Li B., Wang W. (2014). NLRP3 gene silencing ameliorates diabetic cardiomyopathy in a type 2 diabetes rat model.. PLoS One.

[95] Yang F., Qin Y., Wang Y. (2019). Metformin inhibits the NLRP3 inflammasome *via* AMPK/mTOR-dependent effects in diabetic cardiomyopathy.. Int. J. Biol. Sci..

[96] Li X., Li Z., Li B., Zhu X., Lai X. (2019). Klotho improves diabetic cardiomyopathy by suppressing the NLRP3 inflammasome pathway.. Life Sci..

[97] Luo B., Li B., Wang W. (2014). Rosuvastatin alleviates diabetic cardiomyopathy by inhibiting NLRP3 inflammasome and MAPK pathways in a type 2 diabetes rat model.. Cardiovasc. Drugs Ther..

[98] Wang Y., Li H., Li Y. (2019). Coriolus versicolor alleviates diabetic cardiomyopathy by inhibiting cardiac fibrosis and NLRP3 inflammasome activation.. Phytother. Res..

[99] Sun X., Sun X., Meng H. (2022). Krill oil inhibits NLRP3 inflammasome activation in the prevention of the pathological injuries of diabetic cardiomyopathy.. Nutrients.

[100] Zhang H., Chen X., Zong B. (2018). Gypenosides improve diabetic cardiomyopathy by inhibiting ROS-mediated NLRP 3 inflammasome activation.. J. Cell. Mol. Med..

[101] Soares-Silva M., Diniz F.F., Gomes G.N., Bahia D. (2016). The mitogen-activated protein kinase (MAPK) pathway: Role in immune evasion by trypanosomatids.. Front. Microbiol..

[102] Dhiman S., Mannan A., Taneja A., Mohan M., Singh T.G. (2024). Sirtuin dysregulation in Parkinson’s disease: Implications of acetylation and deacetylation processes.. Life Sci..

[103] Zhang W., Liu H.T. (2002). MAPK signal pathways in the regulation of cell proliferation in mammalian cells.. Cell Res..

[104] Avagimyan A., Popov S., Shalnova S. (2022). The pathophysiological basis of diabetic cardiomyopathy development.. Curr. Probl. Cardiol..

[105] Xu Z., Sun J., Tong Q. (2016). The role of ERK1/2 in the development of diabetic cardiomyopathy.. Int. J. Mol. Sci..

[106] Adhikary L., Chow F., Nikolic-Paterson D.J. (2004). Abnormal p38 mitogen-activated protein kinase signalling in human and experimental diabetic nephropathy.. Diabetologia.

[107] Westermann D., Rutschow S., Van Linthout S. (2006). Inhibition of p38 mitogen-activated protein kinase attenuates left ventricular dysfunction by mediating pro-inflammatory cardiac cytokine levels in a mouse model of diabetes mellitus.. Diabetologia.

[108] Wang S., Ding L., Ji H., Xu Z., Liu Q., Zheng Y. (2016). The role of p38 MAPK in the development of diabetic cardiomyopathy.. Int. J. Mol. Sci..

[109] Van Linthout S., Riad A., Dhayat N. (2007). Anti-inflammatory effects of atorvastatin improve left ventricular function in experimental diabetic cardiomyopathy.. Diabetologia.

[110] Fan Z., Dong J., Mu Y., Liu X. (2022). Nesfatin-1 protects against diabetic cardiomyopathy in the streptozotocin-induced diabetic mouse model *via* the p38-MAPK pathway.. Bioengineered.

[111] Wang Y. (2014). Inhibition of JNK by novel curcumin analog C66 prevents diabetic cardiomyopathy with a preservation of cardiac metallothionein expression.. Am. J. Physiol. Endocrinol. Metab..

[112] Marrocco V., Bogomolovas J., Ehler E. (2019). PKC and PKN in heart disease.. J. Mol. Cell. Cardiol..

[113] Newton A.C., Antal C.E., Steinberg S.F. (2016). Protein kinase C mechanisms that contribute to cardiac remodelling.. Clin. Sci. (Lond.).

[114] Singh R.M. (2017). Protein kinase C and cardiac dysfunction: a review.. Heart Fail. Rev..

[115] Geraldes P., King G.L. (2010). Activation of protein kinase C isoforms and its impact on diabetic complications.. Circ. Res..

[116] Giacco F., Brownlee M. (2010). Oxidative stress and diabetic complications.. Circ. Res..

[117] Way K.J., Isshiki K., Suzuma K. (2002). Expression of connective tissue growth factor is increased in injured myocardium associated with protein kinase C beta2 activation and diabetes.. Diabetes.

[118] Lei S., Li H., Xu J. (2013). Hyperglycemia-induced protein kinase C β2 activation induces diastolic cardiac dysfunction in diabetic rats by impairing caveolin-3 expression and Akt/eNOS signaling.. Diabetes.

[119] Connelly K.A., Kelly D.J., Zhang Y. (2009). Inhibition of protein kinase C-beta by ruboxistaurin preserves cardiac function and reduces extracellular matrix production in diabetic cardiomyopathy.. Circ. Heart Fail..

[120] Soetikno V., Sari F.R., Sukumaran V. (2012). Curcumin prevents diabetic cardiomyopathy in streptozotocin-induced diabetic rats: Possible involvement of PKC-MAPK signaling pathway.. Eur. J. Pharm. Sci..

[121] Bagul P.K. (2015). Resveratrol ameliorates cardiac oxidative stress in diabetes through deacetylation of NFkB-p65 and histone 3.. J. Nutr. Biochem..

[122] Valen G. (2001). Nuclear factor kappa-B and the heart.. J. Am. Coll. Cardiol..

[123] Mohan M., Mannan A., Singh T.G. (2023). Therapeutic implication of Sonic Hedgehog as a potential modulator in ischemic injury.. Pharmacol. Rep..

[124] Singh S., Singh T.G. (2020). Role of nuclear factor kappa B (NF-κB) signalling in neurodegenerative diseases: An mechanistic approach.. Curr. Neuropharmacol..

[125] Min W., Bin Z.W., Quan Z.B., Hui Z.J., Sheng F.G. (2009). The signal transduction pathway of PKC/NF-κB/c-fos may be involved in the influence of high glucose on the cardiomyocytes of neonatal rats.. Cardiovasc. Diabetol..

[126] Mazière C., Mazière J.C. (2009). Activation of transcription factors and gene expression by oxidized low-density lipoprotein.. Free Radic. Biol. Med..

[127] Ricote M., Li A.C., Willson T.M., Kelly C.J., Glass C.K. (1998). The peroxisome proliferator-activated receptor-γ is a negative regulator of macrophage activation.. Nature.

[128] Li H., Malhotra S., Kumar A. (2008). Nuclear factor-kappa B signaling in skeletal muscle atrophy.. J. Mol. Med. (Berl.).

[129] Brown K.D., Claudio E., Siebenlist U. (2008). The roles of the classical and alternative nuclear factor-kappaB pathways: potential implications for autoimmunity and rheumatoid arthritis.. Arthritis Res. Ther..

[130] Matsukura S., Kokubu F., Kurokawa M. (2006). Synthetic double‐stranded RNA induces multiple genes related to inflammation through Toll-like receptor 3 depending on NF-κB and/or IRF‐3 in airway epithelial cells.. Clin. Exp. Allergy.

[131] Jia G., DeMarco V.G., Sowers J.R. (2016). Insulin resistance and hyperinsulinaemia in diabetic cardiomyopathy.. Nat. Rev. Endocrinol..

[132] Mariappan N., Elks C.M., Sriramula S. (2010). NF-κB-induced oxidative stress contributes to mitochondrial and cardiac dysfunction in type II diabetes.. Cardiovasc. Res..

[133] Hj L., Yl F., Hh L. (2017). Apigenin alleviates STZ-induced diabetic cardiomyopathy.. Mol. Cell. Biochem..

[134] Alshehri A.S., El-Kott A.F., Eleawa S.M. (2021). Kaempferol protects against streptozotocin-induced diabetic cardiomyopathy in rats by a hypoglycemic effect and upregulating SIRT1.. J. Physiol. Pharmacol..

[135] Levelt E., Gulsin G., Neubauer S., McCann G.P. (2018). Mechanisms in endocrinology: Diabetic cardiomyopathy: pathophysiology and potential metabolic interventions state of the art review.. Eur. J. Endocrinol..

[136] Borghetti G., von Lewinski D., Eaton D.M., Sourij H., Houser S.R., Wallner M. (2018). Diabetic cardiomyopathy: Current and future therapies. Beyond glycemic control.. Front. Physiol..

[137] Murtaza G., Virk H.U.H., Khalid M. (2019). Diabetic cardiomyopathy - A comprehensive updated review.. Prog. Cardiovasc. Dis..

[138] Berezin A.E., Berezin A.A. (2020). Circulating cardiac biomarkers in diabetes mellitus: A new dawn for risk stratification-a narrative review.. Diabetes Ther..

[139] Abdelrahman A.H., Salama I.I., Salama S.I. (2021). Role of some serum biomarkers in the early detection of diabetic cardiomyopathy.. Future Sci. OA.

[140] Kumar M., Dev S., Khalid M.U. (2023). The bidirectional link between diabetes and kidney disease: mechanisms and management.. Cureus.

[141] Pergola V., Cabrelle G., Mattesi G. (2022). Added value of CCTA-derived features to predict MACEs in stable patients undergoing coronary computed tomography.. Diagnostics (Basel).

[142] Tassetti L., Sfriso E., Torlone F. (2024). The role of multimodality imaging (CT & MR) as a guide to the management of chronic coronary syndromes.. J. Clin. Med..

[143] Sperlongano S., D’Andrea A., Mele D. (2021). Left ventricular deformation and vortex analysis in heart failure: From ultrasound technique to current clinical application.. Diagnostics (Basel).

[144] Jm P., Gi V. (2013). Diabetic cardiomyopathy: Pathophysiology, diagnostic evaluation and management.. World J. Diabetes.

[145] Mordi I.R. (2019). Non-invasive imaging in diabetic cardiomyopathy.. J. Cardiovasc. Dev. Dis..

[146] Youssef M.E., El-Azab M.F., Abdel-Dayem M.A., Yahya G., Alanazi I.S., Saber S. (2022). Electrocardiographic and histopathological characterizations of diabetic cardiomyopathy in rats.. Environ. Sci. Pollut. Res. Int..

[147] Maya L., Villarreal F.J. (2010). Diagnostic approaches for diabetic cardiomyopathy and myocardial fibrosis.. J. Mol. Cell. Cardiol..

[148] Sasso F.C., Rambaldi P.F., Carbonara O. (2010). Perspectives of nuclear diagnostic imaging in diabetic cardiomyopathy.. Nutr. Metab. Cardiovasc. Dis..

[149] Kumric M., Ticinovic Kurir T., Borovac J.A., Bozic J. (2021). Role of novel biomarkers in diabetic cardiomyopathy.. World J. Diabetes.

[150] Kodama S., Tanaka S., Heianza Y. (2013). Association between physical activity and risk of all-cause mortality and cardiovascular disease in patients with diabetes: a meta-analysis.. Diabetes Care.

[151] Hordern M.D., Coombes J.S., Cooney L.M., Jeffriess L., Prins J.B., Marwick T.H. (2009). Effects of exercise intervention on myocardial function in type 2 diabetes.. Heart.

[152] Sivasankar D., George M., Sriram D.K. (2018). Novel approaches in the treatment of diabetic cardiomyopathy.. Biomed. Pharmacother..

[153] Vaccaro O., Masulli M., Bonora E. (2012). Addition of either pioglitazone or a sulfonylurea in type 2 diabetic patients inadequately controlled with metformin alone: Impact on cardiovascular events. A randomized controlled trial.. Nutr. Metab. Cardiovasc. Dis..

[154] Grimm D., Jabusch H.C., Kossmehl P. (2002). Experimental diabetes and left ventricular hypertrophy.. Cardiovasc. Pathol..

[155] Deedwania P.C., Giles T.D., Klibaner M. (2005). Efficacy, safety and tolerability of metoprolol CR/XL in patients with diabetes and chronic heart failure: Experiences from MERIT-HF.. Am. Heart J..

[156] Shah A.M., Shin S.H., Takeuchi M. (2012). Left ventricular systolic and diastolic function, remodelling, and clinical outcomes among patients with diabetes following myocardial infarction and the influence of direct renin inhibition with aliskiren.. Eur. J. Heart Fail..

[157] Kawasaki D., Kosugi K., Waki H., Yamamoto K., Tsujino T., Masuyama T. (2007). Role of activated renin-angiotensin system in myocardial fibrosis and left ventricular diastolic dysfunction in diabetic patients--reversal by chronic angiotensin II type 1A receptor blockade.. Circ. J..

[158] Isfort M., Stevens S.C.W., Schaffer S., Jong C.J., Wold L.E. (2014). Metabolic dysfunction in diabetic cardiomyopathy.. Heart Fail. Rev..

[159] Tate M., Grieve D.J., Ritchie R.H. (2017). Are targeted therapies for diabetic cardiomyopathy on the horizon?. Clin. Sci. (Lond.).

[160] Al-Rasheed N.M., Al-Rasheed N.M., Hasan I.H. (2017). Simvastatin ameliorates diabetic cardiomyopathy by attenuating oxidative stress and inflammation in rats.. Oxid. Med. Cell. Longev..

[161] Ewang-Emukowhate M., Wierzbicki A.S. (2013). Lipid-lowering agents.. J. Cardiovasc. Pharmacol. Ther..

[162] Carillion A., Feldman S., Na N. (2017). Atorvastatin reduces β-Adrenergic dysfunction in rats with diabetic cardiomyopathy.. PLoS One.

[163] Jia W., Bai T., Zeng J. (2021). Combined administration of metformin and atorvastatin attenuates diabetic cardiomyopathy by inhibiting inflammation, apoptosis, and oxidative stress in type 2 diabetic mice.. Front. Cell Dev. Biol..

[164] Hayat S.A., Patel B., Khattar R.S., Malik R.A. (2004). Diabetic cardiomyopathy: mechanisms, diagnosis and treatment.. Clin. Sci. (Lond.).

